# A Qualitative Evaluation of a Plate-Method Dietary Self-Monitoring Tool in a Sample of Adults Over 50

**DOI:** 10.1016/j.cdnut.2023.101975

**Published:** 2023-07-20

**Authors:** Celeste C Bouchaud, Justine R Chriqui, May Slim, Jean-Philippe Gouin, Hugues Plourde, Tamara R Cohen

**Affiliations:** 1 School of Human Nutrition, Faculty of Agricultural and Environmental Sciences, McGill University, Macdonald Campus, Ste-Anne-de-Bellevue, Quebec, Canada; 2 PERFORM Centre, Concordia University, Loyola Campus, Montreal, Quebec, Canada; 3 Department of Psychology, Concordia University, Loyola Campus, Montreal, Quebec, Canada; 4 Faculty of Land and Food Systems, Food, Nutrition and Health, Dietetics, the University of British Columbia, Vancouver Campus, Vancouver, British Columbia, Canada

**Keywords:** self-monitoring, adherence, dietary intake, behavior change, plate, plate method, food diary, older adults

## Abstract

**Background:**

Self-monitoring is an important behavioral change technique to help users initiate and maintain dietary changes. Diet self-monitoring tools often involve the itemization of foods and recording of serving sizes. However, this traditional method of tracking does not conform to food guides using plate-based approach to nutrition education, such as the 2019 Canada’s Food Guide (CFG).

**Objective:**

To explore the acceptability, facilitators and barriers of using a plate-based dietary self-monitoring tool based on the 2019 CFG (Plate Tool) compared with a traditional Food Journal (Food Journal).

**Methods:**

The 2 dietary self-monitoring tools were compared using a crossover study design over 2 wk. Adults over 50 (*n* = 47) from Montreal, Canada, were randomly assigned to use one tool over 3 d during 1 wk, then used the other tool the next week. Semistructured interviews (*n* = 45) were conducted after completing the second tool. A qualitative description of the interviews was conducted through an inductive determination of themes.

**Results:**

Facilitators to using the Plate Tool were its simplicity, quick completion time compared with the Food Journal and easiness to use, increased awareness of dietary habits and accountability, with participants expressing that it could help users make informed dietary changes aligning with the CFG. However, barriers to using the Plate Tool were its lack of precision, the participants’ difficulty categorizing foods into the CFG categories and recording intake of foods not present on the CFG.

**Conclusions:**

The Plate Tool is an acceptable dietary self-monitoring tool for healthy adults over 50. Self-monitoring tools based on the plate method should take the barriers described in this study into account. Future studies should compare dietary self-monitoring methods to assess adherence and effectiveness at eliciting dietary behavior change.

## Introduction

Self-monitoring is a technique used to promote behavioral change [[1], [2], [3]]. Multiple reviews [2,[4], [5], [6]] as well as a meta-regression [7] have stated that regular self-monitoring is a key element in lifestyle interventions to improve diet quality [[5], [6], [7]]. First introduced in the 1970s by Kanfer [3], it is considered the first step in self-regulation, an important aspect of the Social Cognitive Theory put forth by Bandura [8] as well as the Carver and Scheier Control Theory [9]. The goal of self-monitoring is for a person to monitor their own behavior [2,3]. Self-monitoring brings about better awareness of one’s behavior in relation to a specific goal and promotes sustained self-regulation toward the attainment of a specific goal [2,8,9]. In relation to nutrition, dietary self-monitoring increases one’s awareness of their dietary behaviors [1], which helps them change their eating behavior to align them with their nutrition goals and thus initiate and maintain positive dietary changes over time [1,2].

The main barrier to the efficacy of diet self-monitoring is low adherence to the tools available, irrespective of the tool used (eg, paper-based, web-based, and mobile) [2]. Ensuring a high level of adherence to diet self-monitoring is key for its efficacy, because engagement with the tool over a longer period of time increases one’s ability to sustain dietary changes [2]. In general, the process of dietary self-monitoring is complex, repetitive, and time consuming and, as such, individuals often stop tracking their dietary intakes 3–5 wk after starting [2,10]. Because the main barrier to self-monitoring adherence is the significant amount of time required to use the tools [2,[11], [12], [13]], moving toward simpler, less burdensome tools to self-monitor diet could help change dietary behaviors long term [12].

The majority of dietary self-monitoring tools have users input portions and serving sizes and are often focused on caloric intake. However, this approach to dietary behavior change no longer conforms with dietary public health recommendations in North America that have transitioned away from portion-based food guides (ie, itemizing food items) to proportion-based recommendations [14,15]. The plate-based method was first developed in the 1970s in Sweden and has been integrated into public health nutrition tools of many countries, including the 2019 Canada’s Food Guide (CFG) [15] and the United States’ MyPlate [14,16,17]. Specific to Canada, the CFG visually depicts the recommended proportion of 3 food groups on a plate (a half-plate of vegetables and fruits, a quarter-plate of whole grain foods, and a quarter-plate of protein foods) without suggesting specific serving targets [15]. The CFG depicts pictures of a variety of different foods in each category, various fruits and vegetables (ie, potatoes, tomatoes, apples, etc.), grain foods (ie, whole grain bread, brown rice, quinoa, etc.), and plant-based and animal-based sources of protein (ie, beef, yogurt, tofu, eggs, nuts, etc.) [15]. These patterns of eating are recommended to help reduce the risk of chronic diseases in the Canadian population by recommending vegetables, fruits, whole grains, and protein foods, with a focus on choosing plant-based protein foods and decreasing processed foods [18].

Importantly, given that there is no itemization of foods in the plate-method, this approach is accessible to all health literacy levels given its simplicity [[19], [20], [21]]. Interventions using the plate-method as a nutrition educational tool have led to positive dietary changes and improved health outcomes for those living with diabetes to manage their glycemia compared with interventions involving carbohydrate counting [19] or calorie counting [22]. Furthermore, this plate-based approach has been found to help those living with diabetes better plan their meals and increase their fruit and vegetable intake [19,23]. Similarly, when used to teach meal planning, this approach has been found to be effective at promoting healthy eating habits and reducing carbohydrate, sugar, and total fat consumption [22,24]. In the nondiabetic population, the plate-method is used as a general public health recommendation by a number of countries [17]. In addition, with age, chronic disease risk increases [25], highlighting the need for self-monitoring tools to be evaluated in this population.

Despite its widespread use as a nutrition educational tool [16,19,22,26,27], to our knowledge, there are no reports having evaluated a dietary self-monitoring tool specifically based on the CFG plate-method. Therefore, the aims of this qualitative evaluation were to explore adults’ perceptions and acceptability of using a paper plate-based self-monitoring tool (Plate Tool) based on the CFG to self-monitor their dietary intakes compared with traditional methods (Food Journal).

## Methods

Participants were eligible if they were aged ≥50 y old, spoke English and/or French, and had access to technology required for video and audio connectivity (ie, Zoom). Those who self-identified as living with a cognitive impairment were ineligible. Participants were recruited through advertisement on a research Centre listserv at Concordia University (Montreal, QC), where ethics was obtained. Interested participants contacted the researchers who screened them for eligibility. A consent form and demographic questionnaire were completed online through the secure survey platform LimeSurvey (Germany, 2017*)*.

**FIGURE 1:**
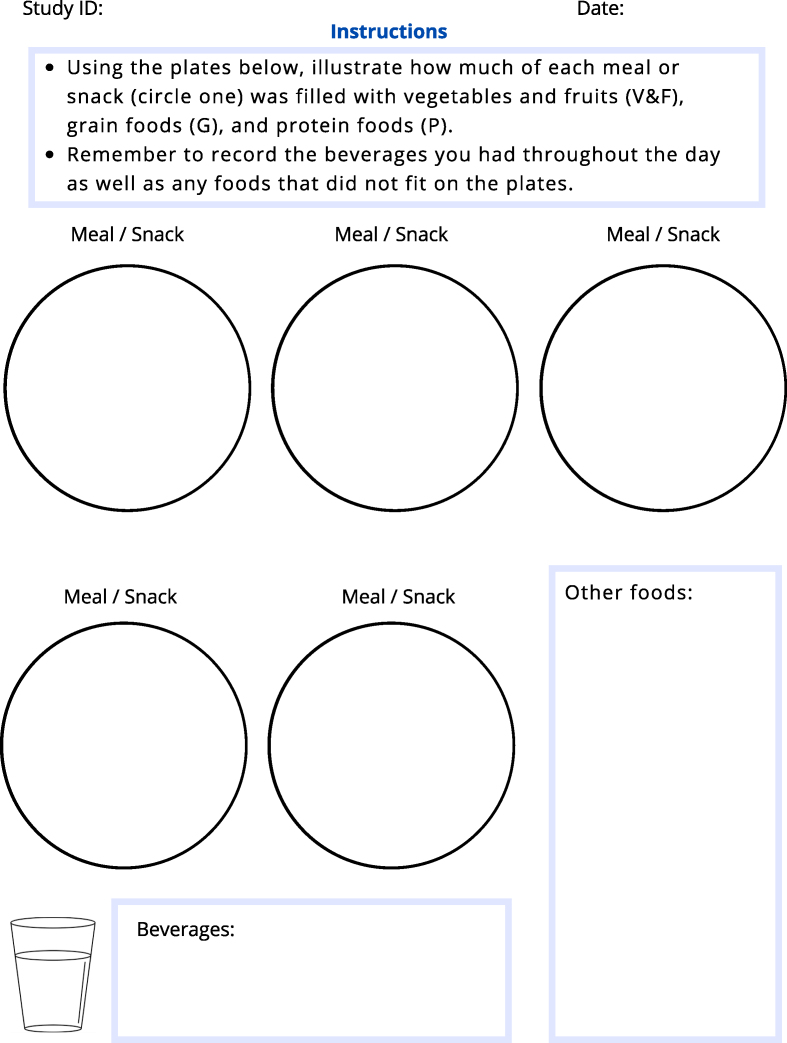
The plate self-monitoring tool evaluated by adults over 50 in the context of this study.

A study package containing paper copies of the dietary self-monitoring tools, instructions on how to use them, and reference materials (portion guide and CFG) [15,28] were sent to their place of residence through postal mail. Once received, a 15–20-min Zoom session with a registered dietitian (CCB or MS) took place to explain how to complete the first tool. Participants were instructed to record their diets >3 d (2 weekdays and 1 weekend day). On completion of the first phase of the study, a second 15–20-min Zoom session was conducted to teach the participants how to use the second tool for the next week. Specifically, participants were randomly assigned to use one tool for 3 d within 1 wk and then instructed to use the other tool for 3 d during the next week. The Food Journal was a traditional food diary where participants were asked to list the quantity and details of all food and beverages consumed, ie, details, such as serving sizes, portions, and brand names, if available. The Plate Tool (Figure 1) was the developed dietary self-monitoring tool based on the CFG [15], which asked participants to illustrate the proportion of their meal or snack composed of the 3 food groups on the CFG [15]: vegetables and fruits, whole grain foods, and protein foods. During the session to explain the use of the Plate, participants were instructed about the CFG [15] and were guided on which foods fit in which category. They were also instructed to write down all “other foods” (ie, processed foods, sources of fat, etc.) which were not represented on the CFG [15] in a separate section on the same page. Beverages were recorded in a separate section. The basis behind the conceptualization of the plate-based dietary self-monitoring tool was based on increasing participants’ adherence to the CFG [15]. Three registered dietitians (CCB, TC, and MS) developed the tool that was used in this study with feedback from a group of undergraduate and graduate students who tested this tool.

As soon as participants had completed both tools, individual semistructured interviews (∼45 min) over Zoom were conducted with CCB, a graduate student or YW, an undergraduate student, to gather data to best capture the participant’s experience using the tools. A semistructured script, pilot-tested and refined within a group of undergraduate and graduate students, with open-ended questions and probes was used to guide the discussions (Table 1). CCB and YW were trained in interview techniques and qualitative methods. Questions were based on the Theory of Planned Behavior [29] to explore participants’ attitudes, subjective norms, and their levels of perceived behavioral control to using the 2 self-monitoring tools. This model was chosen because it would provide insights into behavioral intentions and tendencies to using the tools. In addition, 2 final questions assessed participants’ preference between the tools (“Which method of self-monitoring did you prefer and why?”) and for making changes over time (“Which tool would you see yourself using over time to make dietary changes?”). Participants received a CAD 75 online gift card for completing the study.

**TABLE 1:** Semistructured interview script

Questions about the Journal	Questions about the Plate Tool
Attitudes	Attitudes
• What did you like about the 3-day food journal?	• What did you like about the plate-based tool?
• What did you dislike about the 3-day food journal?	• What did you dislike about the plate-based tool?
• What were the barriers associated with using this tool?	• What were the barriers associated with using this tool?
Subjective norms	Subjective norms
• How did you feel about having to count your portions and fill out this tool throughout the day?	• How did you feel about having to record the proportions on your plate and fill out this tool throughout the day?
Perceived behavioral control	Perceived behavioral control
• What makes it easy to use the 3-day food journal?	• What makes it easy to use the plate-based tool?
• What makes it difficult to use the 3-day food journal?	• What makes it difficult to use the plate-based tool?
Intention	Intention
• How would you feel about using the 3-day food journal to track your diet in the future?	• How would you feel about using the plate-based tool to track your diet in the future?
Perceived benefits	Perceived benefits
• To what extent do you feel that the 3-day food journal would help you maintain a healthier diet?	• To what extent do you feel that the plate-based tool would help you have a healthier diet?
• How do you think the 3-day food journal could help you have a healthier diet?	• How do you think the plate-based tool could help you have a healthier diet?
Overall questions	
• Which method of self-monitoring did you prefer and why?	
• Which tool would you see yourself using over time to make dietary changes?	

### Data analysis

Sociodemographic characteristics were reported as proportions for categorical measures and means and SD for continuous variables using Microsoft Excel (Microsoft Office 2019)*.*

The interviews were recorded, coded to remove all identifiable information, and sent to a licensed transcription service for verbatim transcription (Transcript Heroes-Transcription Services Inc.). All transcriptions were verified by CCB to ensure accuracy of transcription. The transcribed interviews were then imported into QDA Miner 6 (Provalis Research). A qualitative description approach was used to guide the analysis aiming to describe the perceptions and experiences of the participants on using the tools as closely to their experience as possible [30,31].

The transcripts of the interviews were analyzed separately (by CCB and JRC) using an inductive approach [32]: transcripts were read in full, a coding system was developed through the initial analysis of the text, then all comments made by participants were assigned to the codes to generate a final thematic evaluation [33,34]. Themes were consolidated between CCB and JRC, with TC consulted as a third party to resolve any disagreements. Saturation of data was obtained after the coding of the first 15 participant interviews, 2 additional themes emerged through the coding of the remaining interviews.

The final 2 questions were closed-ended questions asking participants whether they preferred the Plate Tool or the Journal or neither. First, asking their preference overall and second, their preference to make changes over time.

## Results

### Characteristics of participants

Forty-seven participants (63.0 ± 5.6 y, 89% Caucasian, 60% female, 78% university educated) were recruited to participate in this study; *n* = 45 completed the semistructured interviews (Table 2) from May to June 2020. Two participants dropped out: one female for medical reasons and one male for unspecified reasons.

**TABLE 2:** Baseline participant characteristics

Variable	All participants (*n* = 47)
**Age (y)**	62.66 (5.75)
**Gender**	
Male	19 (40%)
Female	28 (60%)
Nonbinary or 2 spirit	0 (0%)
**BMI (kg/m**^**2**^**)**	26.2 (4.8)
**Education level**	
University	37 (78%)
College	4 (8.5%)
Vocational or apprenticeship	1 (2.1%)
High School	5 (11%)
**Cultural/racial background**	
White	42 (89%)
Chinese	2 (4.3%)
Arab	1 (2.1%)
South Asian	1 (2.1%)
Black	1 (2.1%)
**Marital status**	
Married or domestic partnership	32 (68%)
Divorced or separated	7 (15%)
Widowed	2 (4.3%)
Single, never married	5 (11%)
**Income level**	
75,000 CAD or more	29 (62%)
30,000–74,999 CAD	8 (17%)
<29,000 CAD	5 (11%)

Values are means (SD) or number (%).

### Overall preferences

When asked about overall preference between the 2 tools, an equal number of participants (*n* = 21) chose the Plate Tool and the Food Journal, respectively, with 3 participants stating no preference (see Table 3). Data analyzed by gender revealed that women primarily preferred the Food Journal overall, whereas men preferred the Plate Tool (see Table 3). When asked which tool they would prefer to use over time to make dietary changes, both men and women stated a preference for the Plate Tool (see Table 4).

**TABLE 3:** Overall preference between the Journal and Plate Tool

Overall preference			
	Both genders (% of total)	Men (% of men)	Women (% of women)
Plate Tool	21 (47%)	11 (61%)	10 (37%)
Food Diary	21 (47%)	7 (39%)	14 (52%)
Neither	3 (7%)	0	3 (11%)
Total	45	18	27

**TABLE 4:** Overall preference between the Journal and Plate Tool to make dietary changes over time

Overall preference for dietary changes			
	Both genders	Men (% of men)	Women (% of women)
Plate Tool	28 (62%)	13 (72%)	15 (56%)
Food Diary	14 (31%)	5 (28%)	9 (33%)
Neither	3 (7%)	0	3 (11%)
Total	45	18	27

### Qualitative evaluation

Words that are *italicized* are themes generated from the thematic evaluation. For a full list of themes related to the Plate, please see TABLE 5, TABLE 6.

**TABLE 5:** Summary of the strengths of the Plate Tool with examples of quotes from participants

Strengths of the Plate Tool	Easy to use	• Easy	“I liked not having to use the scale and follow that, I liked not having to go into the details and this method doesn’t even ask the times, it doesn’t ask the brands, it’s a lot simpler.”
		• Beginner tool	
		• Simple	
		• Sufficiently comprehensive	
		• No quantification	
		• User friendly	
	Quick to use	• Quick	“I think you’d have an easier time using the plate method, over time. Because it’s simpler, it takes less time to fill out.”
		• Higher adherence potential	
		• Versatility	
	Facilitators of the methodology	• Visual	“The groupings were fantastic in terms of, OK, you knew what the grains were, you knew what the protein is, and you knew what fruits and vegetables are. It’s not complicated at all.”
		• Fun	
		• Categorization	
		• Explanation provided	
	Health benefits	• Awareness	“It’s forcing me to eat more vegetables and now after I did my plate thing, I went ‘Oh my god, no grains here’.”
		• Nonrestrictive	
		• Accountability	
		• Inclination to change	
	Reference tool	• Reference to the CFG	“It’s got a built-in goal. Because all you have to do is look at the Canada Food Guide and realize (…) there is something you need to change.”

Abbreviations: CFG, Canada’s Food Guide.

**TABLE 6:** Summary of the challenges of the Plate Tool with examples of quotes from participants

Challenges of using the Plate Tool	Negative elements of the methodology	• Portioning on the Plate	“Understanding the difference between protein, grains and veg, (was difficult) like there are some foods that are not so easy to figure out.”
		• Categorization	
		• Other foods	
	Burden	• Time consuming to complete	“I found that I had to really do it right away after eating (…) at one point I lost the visual in my head of the proportions I had eaten.”
		• Lack of interest in self-monitoring	
		• Remembering to complete the tool	
	Insecurity	• Unfamiliarity	“I found it to be different; I didn’t know this method. It took me a while, in my head, to assimilate it.”
		• Hesitancy	
		• Inadequate instructions	
		• Preference for quantification	
		• Background knowledge required	
	Oversimplification	• Vague	“One barrier was representation because I mean it really is subjective. If I make a pile of lettuce in one corner of my plate that’s six inches high, or it is over the whole plate, who is right, you know?”
		• Subjectivity	

#### The Plate Tool is simple, quick and easy to use

The most cited strength of the Plate Tool was that it was *easy* and *simple* to use. Participants stated that the steps required to use the Plate Tool were *clear* and suggested it is an appropriate *tool for beginners* to use for dietary self-monitoring because it was sufficiently *comprehensive* and contained the necessary level of detail to monitor their diets: “Well it required less detailing, but that was what I liked about it.” Moreover, participants enjoyed the *user-friendliness* of the tool and the *lack of quantification* required when tracking their meals/snacks. Participants liked *categorizing* their intake into the 3 food groups into vegetables and fruits, whole grain foods, and protein foods as depicted on the Plate Tool. For example, categorizing items was “(…) really much easier, because then you're not providing specific quantities exactly, and you're more or less just giving a general breakdown based on 3 types of food groups.” They also felt it was *quick* to complete; participants felt that the tool would have a *higher adherence potential* long-term compared with the Food Journal as its simplicity made it faster to use. Finally, the Plate Tool had an aspect of *versatility* because participants could complete it wherever they were eating (ie, at home, a restaurant, a friend’s house).

#### The Plate Tool’s methodology was novel for participants

Participants appreciated that the Plate Tool was *visual* and was *fun* because they could see the proportions of the different food groups they had consumed at each meal and snack: “I find [it] helps to say I'm eating too much of that food category or I'm eating too much of other stuff.” Whereas many participants, notably women, found the Plate Tool challenging to complete because they were *unfamiliar* with it and were more familiar with itemizing tools, such as the Food Journal. As a result, many participants felt *hesitant* when recording the proportions of their meals on the tool because they were uncertain about whether they were completing it correctly.

Participants had difficulty deciphering what an “*Other Food*” (ie, processed foods, added sugars and fats) was and how to record them. “Flax seeds, chia seeds, we don't know what to do with them. (…) you get the impression that when you put them in other foods, it's as if that doesn't count; it’s not part of the plate.” As a result, participants stated they tended to overlook these foods.

Many participants appreciated the thorough *explanation* that the study dietitians provided because it helped them understand how to best complete their entries. However, some participants shared that they needed more detailed *instructions* on how to correctly use the Plate Tool despite this meeting, including instructions on portioning and categorization of different foods into groups.

#### The Plate Tool has the potential to help users make dietary changes

Many implied that using the Plate Tool would encourage them to eat a healthier diet because it increased their *awareness* about what they were eating. Furthermore, participants reported that the Plate Tool was a *nonrestrictive* way to self-monitor their diet and they did not feel pressured to follow a strict dietary pattern. When using the Plate, many participants described feeling *more accountable* for matching their diet to the recommendations of the CFG and felt *inclined to change* aspects of their diet to meet the proportional guidelines. Many participants also found that the Plate Tool was a useful *reference to the CFG* that could help them follow the new CFG guidelines relating to the proportions of the different food groups in their meal/snack. For example, one participant discussed how the Plate Tool “(…) gives us a more precise idea of how much to eat, protein, fruit, all that. It gives a bit of a guide to what to eat. I will think more about taking grains and protein with each meal, I ask myself the question of whether I have all the groups at each meal.”

#### The Plate Tool lacks precision

Participants stated that one of the main difficulties for using the Plate Tool was the lack of quantification; they would have preferred that the Plate Tool *quantified* their intake, like the Food Journal, because they felt that the information gathered did not tell them enough about their eating habits to be able to alter them. Another barrier was deciding the best way to visually represent the *portion* size of their vegetables and fruits, whole grain foods and protein foods on the 2-dimensional (pen-to-paper) Plate Tool (ie, how to represent 10 almonds at snack-time on the Plate Tool). Participants expressed that the information gained from the Plate Tool *was not accurate* enough; the lack of quantification and itemization prevented them from making concrete changes to their diets. Others described the Plate Tool as *vague* because they found the information gained was too broad to monitor their diet effectively and that the Plate Tool was too *subjective* to determine the proportions and categorization of each food. In the words of one participant: “I think that everybody will have a different idea of what is 20% of my plate.”

Participants also stated they had *difficulty categorizing* some food items into one of the 3 CFG food groups, with dairy products being specified on numerous occasions; participants were not accustomed to their new classification as “protein foods” (as the previous CFG that had a separate food group category for milk and alternatives) [35]. Some stated that a *background in nutrition* was needed to complete the Plate Tool and properly interpret their entries.

#### Both the Plate Tool and the Food Journal have common facilitators and barriers

Participants felt that dietary self-monitoring was a *burden*, irrespective of the tool used, because they found the task overall laborious. Specifically, participants cited it was *time consuming*, and notably men were *not interested in self-monitoring* their diet. In addition, numerous participants reported that they needed to *remember* to complete it and/or physically have the documents and a pen with them at all times, which was a burden. Nonetheless, both tools increased participants’ overall *awareness* of their dietary habits and made them feel *more accountable* when it came to the foods and beverages they consumed.

#### Women and men had different experiences with the tools

Gender differences were noted, with many men stating that they prefer the Plate Tool over the Food Journal because of the latter being too complex compared with the former. Some men mentioned that they were not responsible for the food preparation in their household; they would eat the food that their wife or daughter had prepared. Using the Food Journal meant that they would have to ask their wife or daughter for the ingredients and how the food was prepared, whereas the Plate Tool was easier to complete independently. Women expressed that they were more *familiar* with the Food Journal because they had previously used similar tools in the past.

#### The Food Journal allowed for quantification and detailed food recording although it was laborious and difficult to use

Specifically, participants stated that the Food Journal allowed for *accurate quantification* and provision of *detailed* information about their diets while being *straightforward* and *quick* to complete and the Journal provided a *summary* of the diet. Challenges to using the Food Journal included that it was very *laborious* (some stating that it was *tedious*), and *difficult* to use and requiring many *specifications*. Participants felt this method of tracking was challenging to complete *away from home*, and it is heavily dependent on *memory,* so they needed to *complete it immediately* after their meals or snacks and could lead to *restriction* of their intake. For a list of themes related to the Food Journal, please see TABLE 7, TABLE 8.

**TABLE 7:** Summary of the strengths of the Journal with examples of quotes from participants

Strengths of the Journal	Facilitators of the methodology	• Detailed	“I really liked the detail of it, knowing exactly what I ate. The potato, egg, half a slice of toast, it’s very clear.”
		• Quantification	
	Simplicity	• Straightforward	“It’s pretty straightforward, it’s each meal, just make a list and that’s it.”
		• Quick	
	Familiarity	• Easier over time	“I have that practice already, so this, for somebody who has that practice, or who’s doing, let’s say, Weight Watchers and they’re already journaling things, it’s a very easy transition (to the journal).”
		• Familiar	
		• Matches interest in monitoring	
	Usability	• Goal oriented	“Presumably you’re going to be doing this because you want to achieve some kind of goal, so you would be motivated to do that (…) if you’re doing it out of the blue you would never do this.”
		• Layout of the tool	
		• Summary of intake	
	Health benefits	• Accountability	“It was interesting because it makes you realize what you are eating and not eating. (…) as soon as you start looking at it, you realize that maybe I’m not doing this totally right, or maybe I’m not as holy as I think I am.”
		• Useful to make changes	
		• Awareness	
		• Nonrestrictive

**TABLE 8:** Summary of the challenges of the Journal with examples of quotes from participants

Challenges of using the Plate Tool	Burden to complete	• Laborious	“Whenever I was going to eat something I would always be thinking about how I’m going to write this down and it was (…) weighing on me in a way, thinking about ok, how am I going to describe this? And it’s just annoying to have to get up and go to it and write it all out (…) especially in such detail.”
		• Remembering to complete the tool	
		• Paper tool	
		• Difficult to complete	
		• Lack of interest in self-monitoring	
		• Requires motivation	
	Level of detail required	• Unstandardized portions	“It was the time and effort to write it down and to either figure out what to fill out and checking all the time that I eat the stuff, like, did it really matter?”
		• Specifications required	
	Tool modalities	• Lack of space	“I felt like the piece of paper you gave me, there really wasn’t enough room.”
		• Explanation lacking	
	Interpretation required	• Hard to make appropriate changes	“I’m keeping track, but I could not really make decisions based on what I was writing down. (…) I didn’t see the value.”
	Control	• Restriction	“I might make my portions a little bit smaller but I’m not sure, the more I restrict the more I want to eat. I think this tool encouraged me to restrict.”

## Discussion

This study aimed to evaluate the acceptability of a novel dietary self-monitoring tool based on the plate-method compared with a traditional food journal in a sample of adults over 50. The results of this study suggest that the Plate Tool is an acceptable dietary self-monitoring tool to help adults make positive dietary changes over time compared with a traditional food journal. Participants stated that the Plate Tool was an easy, visual, and quick diet self-monitoring tool that helped them gain awareness of their dietary habits. Using the Plate Tool to self-monitor diet could facilitate positive dietary change and help users adapt their diets that encourage eating according to the plate method, as recommended by the new CFG [15] and nutrition education tools from other countries [14,15,17]. The themes related to the Food Journal were consistent with previous work that food journals are time intensive, laborious, and rely on quantification [2,4,13].

The results of this study align with previous work that aimed to examine if simple dietary self-monitoring tools improve adherence to dietary interventions [12,36]. Previous studies have shown that that simplified tools can improve adherence to self-monitoring, potentially leading to higher adherence and subsequent efficacy [12]. In this study, participants’ preference was for using the Plate Tool over time to make dietary changes compared with a traditional itemization tool, thus potentially increasing adherence to self-monitoring. Furthermore, it has also been shown that there are no significant differences in weight loss between people who used a traditional, detailed calorie- and fat-counting, paper self-monitoring tool compared with an abbreviated paper self-monitoring tool that allowed participants to check mark portion sizes to estimate the fat content and size of their meals and snacks [12]. Participants who completed the abbreviated tool had higher adherence to self-monitoring and both groups had similar levels of weight loss [12]. As such, the proposed plate-method approach to dietary self-monitoring does not only have the potential to be less of a burden on the user, but could ultimately increase adherence to self-monitoring, leading to changes in dietary intake [12]. Future work is needed to test these hypotheses.

Indeed, although the Plate Tool used in this study was deemed to be a simple tool overall, there were some unique challenges associated with the use of this self-monitoring tool. Many foods, such as fat sources (ie, butter, margarine, oils, salad dressings, etc.) and processed foods are not represented on the Plate Tool because they are not depicted on the CFG. Not having a space for these foods on the literal “plate” can affect the interpretation of the users’ diets because these foods significantly increase energy intake. Thus, further research is needed to determine how to best represent these foods while maintaining the overall simplicity of the tool. The same holds true for highly processed foods. Participants also expressed difficulty with categorizing foods in the 3 groups highlighting the need for education to teach users how foods should be classified.

Research has shown that gender plays a role in dietary intake [37]. A strength of this study was our ability to recruit 40% of our sample who identified as men. Gender, as a social construct, affected perceptions of our participants of using the Plate Tool. In this study, more women preferred the Food Journal, the traditional tool, compared with men. In general, women are more interested in healthy diets compared with men, are typically responsible for food preparation [38], and have a greater likelihood of having dieted in their lifetimes [37]. Although we did not specifically survey for dieting history or history of diet self-monitoring, our analysis resulted in women reporting more familiarity with the Food Journal through previous dieting experiences. As traditional dietary self-monitoring tools, notably calorie counting has been linked to eating disorders [39,40], the Plate Tool may be an appropriate tool to provide to users to limit this, allowing those living with eating disorders to monitor their diets without promoting detrimental eating habits [41]. Many women interviewed in this study stated that they had previously trialed various itemizing self-monitoring tools, and therefore it was not surprising that a small majority of women in this study chose the Food Journal when asked which tool they preferred overall. However, when asked about which tool they would prefer for dietary self-monitoring over an extended period of time, a small majority of women preferred the Plate Tool, despite their familiarity with the Food Journal.

In contrast, most men stated that they would prefer to use the Plate Tool to self-monitor their diets, with the majority stating that they prefer to use it over an extended period of time to facilitate long-term dietary changes. Men expressed less familiarity with the Food Journal compared with the women in the sample and expressed that they would prefer using the Plate because it was simpler and they would not have to rely on a female member of their household to tell them what the specifics of their prepared foods to be recorded. Although we did not survey dietary history or ask participants about how or who prepares the homemade meals, these findings could be related to men having an overall lower prevalence of dieting history compared with women [37] and women being responsible for the majority of food preparation in the household [38]. Although limited to the gender binary, these gender differences could have implications on which self-monitoring tool to choose when designing interventions for people of different genders.

### Strengths and limitations

Although this study suggests that the Plate Tool could be a self-monitoring tool used to promote dietary behavior change that is aligned with the CFG, limitations to using this method exist. The simplicity of this tool does have drawbacks, such as the inability to calculate macro- and micro-nutrient intakes. However, this Plate Tool was not designed to be a specific dietary assessment tool but as a global self-monitoring tool to encourage meal planning and promote positive dietary changes over time. Second, the Plate Tool is based on the CFG, a public health tool intended to be a generalized recommendation for the entire Canadian population, and therefore does not consider the needs of specific populations living with chronic diseases. The instructions provided to the participants to complete the CFG were limited to what had been published at the time by the government [15]; thus, no list of foods existed that categorized foods into different categories, limiting the comprehensiveness of the instructions provided to participants.

In addition, we did not examine actual adherence (ie, days completed) to using the tools. Our sample was also homogeneous; we recruited healthy adults over 50, primarily Caucasian, highly educated, and from high socioeconomic status, limiting the generalizability of our findings. Higher education status and higher income have been consistently associated with more health promoting diets [42], lower levels of food insecurity [43], and higher health literacy levels [44], leading to different experiences with the tools used, further limiting the generalizability of the results presented in this evaluation. It will be important to evaluate the use of the Plate self-monitoring tool in a representative sample of Canadians.

### Conclusion

In conclusion, the Plate Tool is an acceptable and user-friendly method to self-monitor diet in adults over 50. This tool has the potential to elicit positive dietary changes and increase adherence to eating according to the CFG compared with traditional dietary self-monitoring tools, such as food journals. This study also highlights that further research is needed to determine how to include certain food types, eg, highly processes foods, while maintaining the overall simplicity of this self-monitoring approach. Future studies should test the acceptability of using the Plate Tool in different cultures and ages and test the feasibility of using this tool over time to determine if its use is related to positive dietary changes.

## Acknowledgments

We acknowledge Yolanda Wang for helping the team conduct the semistructured interviews.

## Author contributions

The authors’ responsibilities were as follows – TRC, MS, HP, J-PG, CCB: designed research; CCB, MS: conducted research; CCB, JRC: analyzed data; CCB, JRC: wrote the paper; TRC, MS, J-PG, HP: reviewed the analysis and the paper; CCB: had primary responsibility for the final content; and all authors: read and approved the final manuscript.

## Conflict of Interest

The authors report no conflicts of interest.

## Funding

The study was funded by R. Howard Webster Foundation.

## Data availability

Data described in the manuscript, code book, and analytic code will be made available upon request pending application and approval.
